# The Meaning of Leprosy and Everyday Experiences: An Exploration in Cirebon, Indonesia

**DOI:** 10.1155/2013/507034

**Published:** 2013-03-20

**Authors:** Ruth M. H. Peters, Dadun, Mimi Lusli, Beatriz Miranda-Galarza, Wim H. van Brakel, Marjolein B. M. Zweekhorst, Rita Damayanti, Francisia S. S. E. Seda, Joske F. G. Bunders, Irwanto

**Affiliations:** ^1^Athena Institute, Faculty of Earth and Life Sciences, VU University Amsterdam, De Boelelaan 1085, 1081 HV Amsterdam, The Netherlands; ^2^Centre for Disability Studies, Selo Sumarjan Research Center (SSRC), Faculty of Social and Political Sciences, Universitas Indonesia, Gedung H, 6th Floor, Cubicle E, Kampus FISIP UI, Depok 16424, Indonesia; ^3^Center for Health Research, Faculty of Public Health, Universitas Indonesia, Gedung G, R. 211, Fakultas Kesehatan Masyarakat, Kampus UI, Depok 16424, Indonesia

## Abstract

It is imperative to consider the meaning of leprosy and everyday experiences of people affected by leprosy and key persons in the community if one aims to make leprosy services more effective, which appears necessary in Indonesia given the large numbers of new cases detected annually. However, little is written in the international literature about the experiences of people currently being treated for leprosy, those cured, or other key informants. This paper analyses the narratives of the people by drawing upon in-depth interviews with 53 participants and 20 focus groups discussions. The participants were purposively selected. We provide insights into the experiences of people and the meaning they give to leprosy and highlight aspect of aetiology, spirituality, religion, darkening of the skin, and sorcery. We also examine experiences of seeking care and focused on the impact of the disease in particular on the elderly and children. In conclusion, the continued need for implementation of leprosy services in Indonesia is very evident. The diversities in people's experiences with leprosy indicate a demand for responsive leprosy services to serve the diverse needs, including services for those formally declared to be “cured.”

## 1. Introduction

Over the last decades, efforts in the field of leprosy have focused on curing the disease, controlling its spread, and preventing impairments. From a global perspective the results are impressive and commendable with a decline of the number of new cases and the reduction of the proportion with severe visual impairments at diagnosis [1]. For some countries, among others Indonesia, the picture is not yet so notable. Also for implications beyond the medical scope such as stigma, the road is still long [2–4].

Leprosy has been associated with stigma throughout history [5–9]. Manifestations of stigma, including self-stigma, social exclusion, and discrimination, although nowadays more subtle with less ostracism, remain a reality for many people affected [10]. To help leprosy services become more perceptive towards issues surrounding leprosy-related stigma and reduce its impact, it is necessary to understand stigma from the perspective of the people affected and their family members. Also the views of key persons in the community, such as neighbours, teachers, religious leaders, and health workers, should be considered. “The human face of leprosy” edited by Gokhale and Sohoni [11] in 1999 already emphasised the need for such stories.

Several studies related to leprosy have been executed in Indonesia focussing on biomedical aspects [12–14], risk factors [15–17], case finding [18, 19], and gender [20]. Although these studies on leprosy are valuable in their own discipline, we identified a lack of knowledge in the published international literature on the experiences of people currently undergoing treatment or already cured and other key informants in Indonesia and elsewhere. 

Hence, the Stigma Assessment and Reduction of Impact Project (SARI), of which this study is a part, decided to undertake an exploratory study into these experiences and perceptions, prior to starting a participatory process of designing and implementing stigma-reduction interventions. The main research questions of this study were how is leprosy understood and experienced in people affected by leprosy and key persons in the community and what implications do these understandings of leprosy and experiences have for leprosy services?

This paper starts with a brief overview of leprosy control and epidemiology followed by a concise analytical framework. After the materials and methods, this paper highlights the human face of leprosy behind the statistics and tries to do justice to the diversity of experiences and the local belief system in the study area. In line with this, our results will be presented according to five main themes that emerged from the analysis: (i) giving meaning to leprosy, (ii) aetiology, (iii) seeking care, (iv) understanding healing and cure, and (v) impact of leprosy. The views and experiences of people with leprosy and other key persons in the community members are compared, underlining those that are relevant for the improvement of leprosy services and reduction of stigma. 

## 2. Brief Overview of Leprosy Control and Epidemiology in Indonesia

Indonesia presently has the third highest level of leprosy infection in the world, after India and Brazil [1]. Twelve, of the in total thirty-four, provinces in Indonesia have new case detection rates above 10/100,000 population, West Papua has a rate above 100/100,000. Indonesia has a long history of leprosy control. In 1655, the first leprosy asylum was built on one of the islands in the bay of Jakarta and over two centuries the number of asylums increased until 45 [23]. In 1932, the compulsory isolation of leprosy cases was abolished [23] and the implementation of several national control programs followed. Already in 1969, the government started to integrate leprosy control in the general health services [24]. Currently, the second strategic plan for the National Leprosy Control Programme (NLCP) 2011–2015 is implemented [25].

**Figure 1:**
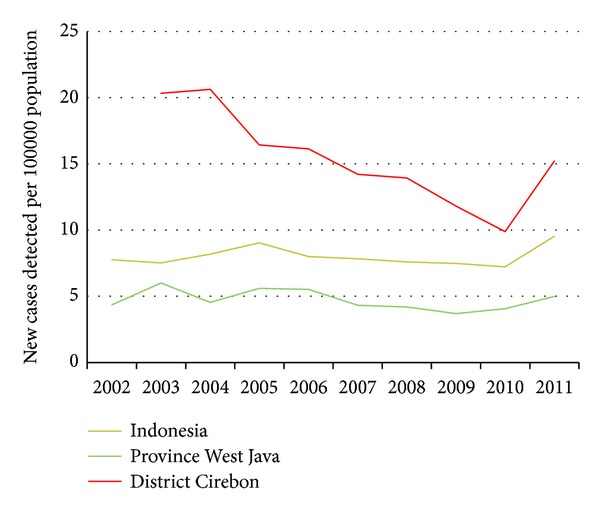
10-year trend of new case detection rate in Indonesia, West Java and Cirebon in 2002–2011 (data is merged from [21, 22]).

We will focus on the epidemiology of leprosy in Cirebon district, as this is the area of research for this study and put the numbers in the context of the provincial (West Java) and national figures. Last year, the District Health Office of Cirebon reported 320 new leprosy cases (15.2/100,000 population). As shown in Figure 1, the number of new leprosy cases on national level showed a flat pattern until 2010. Last year, the Ministry of Health of Indonesia reported 23,000 new leprosy cases (9.5/100,000 population); an increase of 35% compared to 2010. We are not aware of any recent changes in the leprosy services that can account for this increase. Part of the explanation for the increase at the national level could be increased case detection activities conducted by the NLCP, particularly in some high-endemic and/or remote and difficult to reach areas. 

**Figure 2:**
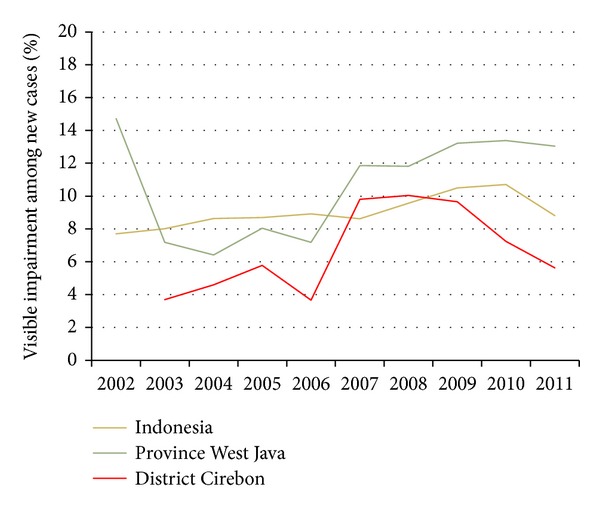
10-year trend of % visible impairments among new cases in Indonesia, West Java and Cirebon in 2002–2011 (data is merged from [21, 22]).

Of the new cases in Cirebon, 18 (5.6%) had visible impairments (also referred to as “grade-2 disabilities”); this percentage has been gradually increasing over the years, but decreased slightly since 2009. Similar trends can be seen on national and provincial level see Figure 2. The figures suggest a rather late case detection, but the reduction is promising and indicates that the current leprosy control programmes are making progress. 

**Figure 3:**
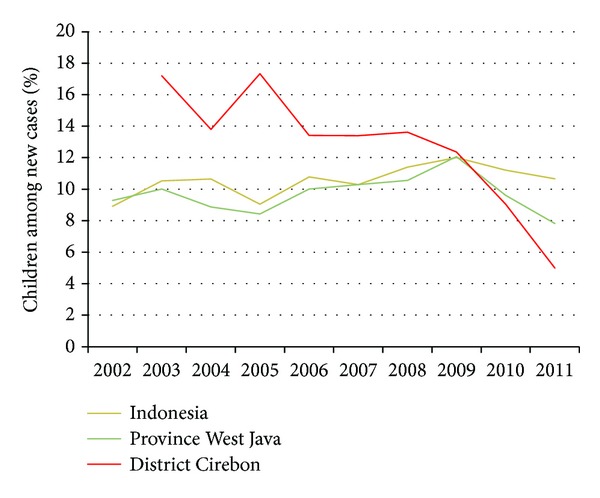
10-year trend of % children among new cases in Indonesia, West Java and Cirebon in 2002–2011 (data is merged from [21, 22]).

Figure 3 shows a varying, but declining percentage of new cases among children particularly in Cirebon. This indicates progress in the reduction of recent leprosy transmission. 

Data on the percentage of women among new registered cases was only available from 2008 until 2011 at national level. After an initial increase, the number has declined from 39.8% in 2009 (6,877 cases) to 34.6% in 2011 (7,968 cases). 

## 3. Analytical Framework

The experiences of people affected by leprosy are conceptualized in diverse ways. Concepts of social exclusion, discrimination, and stigma are frequently used. In particular for the concept of stigma, several conceptual frameworks have been developed [5, 26–31], often taking Goffman's now classic work on a “spoiled identity” [32] as point of departure. One commonly used conceptual framework is the one of Weiss [26]. Weiss extended the Hidden Distress Model of Scambler [28] and distinguishes six types of stigma, three from perpetrators and three from those who are stigmatized. Perpetrators exhibit accepted, endorsed and enacted stigma; the latter is often called “discrimination.” Those being stigmatised exhibit anticipated (or perceived), internalised (or self-stigma), and enacted (or experienced) stigma [28, 33]. To make productive use of our results and, in particular with future leprosy services and interventions in mind, we use conceptualizations of Weiss where appropriate.

## 4. Materials and Methods

### 4.1. Study Area

Cirebon District located on the North Coast of West Java, was selected as the area of research and project implementation because it has a high number of new cases annually and has accordingly local experts higher leprosy-related stigma in comparison to other districts. Cirebon has a vibrant history and not surprisingly, is known as a cultural melting pot; it has absorbed influences from Hindu, Buddhist, Islamic, Sundanese, Javanese, Chinese, and Dutch cultures. This is also reflected in the name Cirebon (originally Caruban) what literally means “mixed”. 

### 4.2. Research Team

Six research assistants assisted the four main researchers with conducting interviews and focus group discussions. The research assistants all come from Cirebon district and speak the local languages Sundanese, Javanese and Cirebonese, in addition to the national language, Bahasa Indonesia. Some research assistants have either a disability or have been affected by leprosy themselves. They received training in social research (1 week) and community-based rehabilitation (3 weeks). During data collection, meetings with the whole team were held at the office to share experiences, challenges, feelings and stimulate learning. These meetings were initially held daily and later on weekly basis. This was done to develop a common understanding of the local situation, build a strong connection with the research, improve research skills, and improve the approach.

### 4.3. Sampling and Selection

The *puskesmas* (Community Health Centres) have provided the contact details of people affected by leprosy. The research assistants have played an important role in updating the list, identifying potential participants, and inviting them to be part of the study. The participants were purposively selected, based on characteristics such as age, gender, religion, and role in the community. 

### 4.4. Data Collection Methods

To understand the variety of experiences of people currently under treatment for leprosy or already cured, interviews were conducted in June and July 2011. Each participant was interviewed three times. This was done to build trust and help participants feel confident to talk about the issues they faced in daily life. In total, 53 (times three) interviews were conducted, of which nine were with children. The interviews were conducted with single participants and in pairs of interviewers. The interviews commenced in an exploratory manner and then progressed towards more in-depth enquiry. Topics addressed in the interviews were (i) general information, (ii) life history, (iii) economic situation, (iv) social situation, (v) health situation, and finally (vi) leprosy. In addition, different visualisation techniques were used. For example, body maps were employed to investigate the participants' perception and implicit knowledge of their own bodies and the connection with the disease. This made it possible to interpret the aetiology of leprosy. Photographs of the families were used to open an intimate space for a dialogue about family issues. In addition, throughout the data collection period, several informal interviews took place; these were also considered during the analyses.

To understand the perspective of the community, twenty focus group discussions (FGDs) were conducted. Participants were neighbours of those who had leprosy, community and religious leaders, mothers of children affected, teachers, and health workers. Each FGD had between 4 and 12 participants. The FGDs were conducted in June and July, October and November 2011. The majority of FGDs took place in the SARI office but, when it was considered to be more appropriate, a meeting room in the District Health Office or a local hotel was arranged. Each FGD was designed slightly different, but the purpose was the same: to collect information from different informants about the situation of people affected by leprosy and their families in Cirebon district. The common themes were (i) understanding of leprosy, (ii) stigma in the community, (iii) main issues in the community, (iv) current strategies, and (v) recommendations for strategies to reduce stigma. A community map was chosen as visualisation technique to explore the main problems that affect their communities in general and specifically related to leprosy and the relationships among them.

The results presented below draw on these interviews and FGDs, but do not represent a complete analysis of the available data. The interviews and FGDs were recorded, transcribed, and translated into English. The transcripts were entered and analysed in QSR NVivo 9. First, data was coded and memos were made to find themes, clusters, and patterns. To organize, compare, summarize, and finally draw conclusions, several models were formed. 

### 4.5. Ethical Considerations

Permission for the study was approved from the relevant government offices. Written consent was obtained from individual study subjects. Incentives, such as travel expenses, were refunded, lost earnings compensated and/or sometimes a small present (t-shirt, mug) was given to the participants as a token of appreciation. 

## 5. Results

### 5.1. Giving Meaning to Leprosy

Two terms, *lepra and kusta* were commonly used by our informants to designate leprosy which has been confusing for many participants. The first is derived from India where leprosy most probably originates from. The name “Kushtha” was derived from “Kushnati” which is believed to mean “eating away” in Sanskrit [34]. The second word *lepra*, is derived from the Greek word Λ*έπρα* [léprā or lepros] and was used by physicians to refer to a scaly skin disease [4]. Some participants believe that the two terms mean the same, while others believe that these terms identify a difference in the severity of the disease. Several participants were of the opinion that there are different types of leprosy. Sometimes a connection is made to diabetes; one community leader even stated that leprosy and diabetes are the same (FGD 2 community leaders). 

Leprosy as a health issue is understood in different ways, which is illustrated by the variety of answers given to the question: “What is leprosy?” Participants often initially responded that they did not know what leprosy is and some questioned the interviewer. For example, they said “I want to ask … Is it true that leprosy can affect children genetically?” (FGD 2 community leaders) or “I heard that all of their fingers will come off. Is that true?” (Interview 7: female, age 36). 

When participants shared their views about leprosy, they often referred to it as a skin disease, emphasising the contagious nature of the disease and impairments of hand and feet. They described the characteristics of leprosy as follows: “red or white rash,” “face got red,” “lumps,” “swollen ear,” “this vein … is hard,” “you cannot feel anything when you are pinched,” “feet a little bit open,” “lesions,” “skin will peel off,” “hands shrank,” and “permanently damaged body function.” Additional symptoms that were frequently mentioned by people who had suffered from leprosy themselves were “hot,” “itchy,” and “pain.” A teacher compared leprosy to a ripe mango.

*I thought the lesions had become as ripe as fruit. The rash was pretty ripe in my opinion; it's just like the black freckles on ripe mango. (FGD 6 teachers)*

Interestingly, sometimes concepts as shame and low self-confidence were perceived symptoms indicative for having leprosy, as shown by the following quotes.

*As far as he knows about leprosy: the face become pale, become introvert and did not want to join in their activity. (Interview 13: male age 62)*

*I am not shy and I do not have a low self-confidence, so I do not have leprosy right? (Informal interview 1: male)*

What also stands out in terms of characteristics is the darkening of the skin as a side effect of the multidrug therapy. People affected by leprosy said that sometimes it is the dark skin and not so much the leprosy that makes them feel inferior. Also, the dark skin triggered friends and neighbours to ask questions which made people affected feel uncomfortable. One participant lied during job interviews by saying that he loves playing kite and that his skin is dark because of that. As a side note, in general in Indonesia a lighter skin is perceived to be more beautiful or “cleaner” than a darker skin as also illustrated by the response from a mother whose son was affected by leprosy.

*I was surprised because he [her son] became so dark-skinned. He was so clean before, but he became so dark. … He was so dark, really really dark. (Interview 3: female age 45)*

### 5.2. Aetiology

The reported ideas on aetiology vary. Firstly, participants think it is an infectious disease that can easily spread through direct contact with the person affected. This also implies that, in their perception, breathing the same air, shaking hands, eating food prepared by a person affected, carrying the deceased body of a person affected or using the same personal objects, such as glasses, towels and clothes, could potentially transmit the disease. However, this perspective is not shared by all participants as some think it is not contagious. Participants also referred to the importance of having the same blood or same blood type, some consider this to be a prerequisite for infection. Three related quotes are as follows.

*Mother: I used to be free from the disease but I got infected through my neighbour. *

*Interviewer: Ooo, I see.*

*Mother: Yes..it is my mistake… He keeps coming to my house to play, so the disease infected us. (FGD 4 mothers of children with leprosy)*

*Teacher: Even in Eid Mubarak we do not shake hand…*

*Interviewer: So when you meet a patient of leprosy, and you know that the person affected by leprosy, you will not shake their hand when they want to?*

*Teacher: Not really. Well, coincidentally, the person always keeps himself inside the house… he understands it somehow. (FGD 6 teachers)*

*Leprosy is like….like what I have. It does not seem contagious. If it were contagious, my children would be affected. (Interview 9: male age 36)*

The next category is other biological reasons such as heredity: some believe this is the case as they have seen other members of the same family suffering from leprosy. Others mentioned through “breastfeeding the baby,” derived from the observation that the baby's skin can turn darker if the mother who is breastfeeding is still on treatment. The third category of causes relates to poor hygienic conditions such as swimming in a dirty river. The fourth refers to “logical explanations” related to certain activities. For instance, since some of the people affected work with goats, work in construction or have eaten chicken, a link between the animals, the cement, or eating chicken has been used to explain the cause of leprosy. The final category refers to the supernatural and moral aspects of life. Many people affected by leprosy believe leprosy is a challenge from God. People thought that God could be involved in allowing the bacteria to attack the body of the person, but with the purpose of making them stronger internally and in their faith. In contrast, a moral cause is involved when human deeds are seen as a reason for contracting leprosy. Some key persons in the community perceived leprosy as a punishment from God. A specific example mentioned as a cause for leprosy was having sexual intercourse with a woman while she has her period. Destiny was also mentioned, for example by a teacher, who said he knew persons who passed away because of leprosy, but also clarified that death comes because it is written and hence should be perceived as destiny. In some cases people believe sorcery is involved. These thoughts on aetiology do not exist in isolation: people could believe that there is a divine will but at the same time, they know that there is a scientific explanation. A quote to illustrate this point:

*I think an illness is divine will. But there is a cause of an illness. (FGD 5 religious leaders)*

### 5.3. Seeking Care: Perspectives on Diagnosis and Treatment

Most participants affected by leprosy have consulted the community health services (CHS) for their health problems. A few noted that they faced barriers to reach the CHS. Two participants for instance needed medical treatment but did not have enough money to pay the public transport from their house to the CHS. 

In the CHS some doctors or leprosy workers did not immediately diagnose leprosy but for instance thought the person was suffering from “scabies,” “a sweat allergy,” or “a skin fungus disease”. When the disease did not get better, participants returned to the clinic to find out they were actually suffering from leprosy. Likewise, leprosy workers shared that also people affected by leprosy sometimes underestimate the severity of the symptoms for example they think it is “only wormhole” and as a result delay a visit to the clinic (FGD 20 Leprosy workers). 

Some doctors and leprosy workers diagnosed the disease correctly, but decided not to share the diagnosis with the patient. This happened, for example, with a 29-year-old woman (interview 43). During her consultation the doctor told her she was suffering from a general skin disease, despite that she kept asking, the doctor did not want to tell her more but advised her to take free medicine every day for one year and to collect it monthly at the CHS. Other leprosy workers decided to share the diagnosis, but participants regularly preferred to hide their disease from neighbours, friends, family members, and in some cases their spouse. They also ask leprosy workers to be discrete. Nevertheless, this does not automatically mean people around them did not find. 

The initial encounters between a doctor or leprosy worker and newly diagnosed patient are important. Stigmatizing attitudes and behaviour of doctors and leprosy workers can have an enormous impact on the people affected as illustrated by the first quote. The second quote of a leprosy worker confirms that several health workers are afraid to contract the disease.

*The moment the leprosy worker did not want to shake my hand, I had the feeling leprosy cannot be cured and that people will not be friendly with me anymore. (Informal Interview 2: male) *

*The health worker still feels afraid, nervous …, actually there are still many [health workers] that feel so… (FGD 12 Leprosy workers).*

The CHS usually executes a certain procedure that includes a contact survey (finding new cases) and socialisation (raising awareness) when a new leprosy case is found. The importance of pictures and the use of simple and the right local language were emphasized by a leprosy worker. Another one described the procedure:

*If we find the leprosy patient, we usually come to their house and deliver understanding to them or to their family as well as the society around them … So it is describe on what is leprosy and how to cope with it…that is we usually do in the field… (FGD 12 Leprosy workers)*

An older woman (Interview 18: age 74), however, felt disappointment with the socialising procedure of the leprosy worker as it made neighbours actually more afraid and as a result they avoided her. In contrast, some persons affected by leprosy said they benefited from these visits (Interview 1: male age 20, Interview 45: male age 21, Interview 3: female age 45). They said they were visited routinely at home by leprosy workers who gave counselling to increase confidence and to provide information to their family, who supported them in daily and community activities. 

Several participants sought assistance outside the CHS. Some went first to a pharmacy for general medicine, which is common practice according to a participant. Some went directly to a hospital for which assistance from the head of the village is needed. In addition, several participants went to a *dukun* who can fulfil the role of traditional healer, spirit medium and occasionally sorcerer. Reasons that were given were curiosity, believing that the cause is sorcery and accessibility. One participant did not gain from it as illustrated by the following quote.

*Some friends advised me to go see a dukun… There is one neighbour … took me to see a dukun …, I forgot her name, she is not solving or healing my illness but asking for more money. Since then I never went to dukun again. (Interview 18: female 74)*

Some participants deny the disease and are quite persistent in this; hence they were also reluctant to seek care. One community leader explained that it is because they feel embarrassed and are afraid for negative responses from others in the community as illustrated by the following quotes.

*Based on my first experience, someone suffers from this illness and until right now she is been denying it as leprosy. She was asked to go to the hospital but she did not want to go. And one day people from Health Department came to her house and she still with her persistence. (FGD 20 Leprosy workers) *

*One of the affected people does not want to admit that he suffers from the disease even though his fingers come off. He does not want to find any medication. He is very ashamed. (FGD 2 Community leaders)*

*But if one gets leprosy, rumour has it; they are reluctant to have treatment. I once asked …“have you consulted to the health centre?” and he answered, “No way. People will be looking at me.” He feels so much embarrassed. … Even though he is not sure whether he has leprosy … He started to keep himself away from the neighbour even they had not known at the moment. … (FGD 2 Community leaders)*

### 5.4. Understanding Healing and Cure

Different words are used by the people affected by leprosy to describe that they are “cured”, “healed” or “cleaned”. Several participants emphasize the importance of God in a cause of the disease as described earlier, but also in getting cured as shown by the following quotes.

*Every disease has its remedy and it depends on God's mercy. (Interview 28: female 20)*

*Well, if God listens, then my prayers will be granted and it means I am healed. (Interview 29: male age 36)*

Some people affected clearly indicated that even after being declared “cured”, doubts remain. Some, for example, want a second opinion and others constantly keep an eye on their body or the body of the family member. Interesting are the interviews where the participant, also the ones without impairments, say in the beginning state that s/he is cured but when the interview continues and the interviewer asks about their hopes for the future, indicate the hope to be cured. 

### 5.5. Impact of Living with Leprosy

Leprosy made people affected live with spots, scars, a dark skin and sometimes sensory loss and physical deformity. Some participants as a result refer to their “broken body.” Moreover, some of the participants experienced leprosy reaction after declared cured which caused pain, cramps, and/or paraesthesia that made them continue feeling bad. All of those physical impacts challenged their life and also influenced their emotions and social and economic situation.

Sadness, frustration, loss of confidence, devaluation of their own capacity, stress, and hopelessness were some of the emotions described due to the leprosy. A few people affected told us that they have considered ending their lives. A teacher describes it as follows.

*If they have a high spirit to survive, undergoing treatment, they will live longer… But if they keep hiding and staying away from the sunlight, it is possible for them to get depressed and die… because of depression. (FGD 6 Teachers)*

We noted an interaction between self-isolation and being isolated by the community. Several people affected became reserved, shy, and ashamed and isolated themselves, but at the same time, several family members and people in the community also isolated people affected. Key persons in the community have a variety of views about what is the cause of isolation.

*It is like anyone with this rare disease. People do not really isolate that person but it is that person who isolates him/herself from the society. Most of the people with leprosy tend to act that way. (FGD 6 Teachers)*

*Interviewer: Do all people isolate the affected?*

*Community leader: Well, yeah. People affected are usually isolated and I feel sorry about it. (FGD 2 Community leaders)*

We tried to understand the reasoning behind isolating somebody. We heard quite shocking examples of isolation: a child lived, ate, and slept in a separate room, spouses that do not sleep together anymore and a mother who distanced herself from her children. However, often there are underlying reasons of protection and care illustrated by the following quote.

* Interviewer: What will you do if you are affected by leprosy?*

*Woman: I will isolate myself. … Yes, because I do not want to infect my children. (Interview 7: female age 36)*

The economic situation of the persons affected and their family deteriorates. Some participants were physically not able to do the work that they used to do such as making furniture. Some were fired because of leprosy such as a cleaner in the hospital, while others resigned themselves as suggested by family members. Furthermore, people in the community avoided being customers of street vendors, in *warung* (small restaurant) or small shops, because of fear of being infected by touching the objects or eating the food that is sold by persons affected by leprosy. A community leader said that also farmers face problems, because their feet stand in the same water during rice planting and people are afraid to be infected. The socioeconomic status of the household in effect influences for example other activities such as education. 

The impact can remain for a long time, even after being declared cured. One participant (Interview 35 male, age 41) who has impairment due to leprosy said that most people more saw him as a body rather than as a human being. However, also some participants without any visible signs are affected for a long time. Some shared to have remained feelings of shyness and limited motivation to participate in social activities. 

Elderly and children are important subgroups but are often forgotten. The elderly affected by leprosy interviewed have expressed feelings of sadness because of the lack of care they receive. Their situation is often aggravated by the absence of their family either because most of their relatives have died or because they were rejected when the leprosy started. Moreover, issues as loneliness, dependence on others, other health issues and often an alarming financial situation kept surfacing during our conversations with the elderly. 

Schooling is highly valued in Indonesian society, but the majority of the children affected by leprosy stopped attending school temporarily or permanently. Several reasons to discontinue school were mentioned and these can be categorized into feeling (i) embarrassed, ashamed and shy, (ii) peers making fun of them and taking distance, and (iii) fear of being insulted. 

Some of the respondents have said that it is impossible to think that they would be only sad or only happy all the time; there are some good moments accompanied by some other that are not so good. “Leprosy has made us stronger,” one of them said. When the disease has been cured, there is also the feeling of being strong enough to overcome problems. Several participants have expressed they have gained strength from their spiritual beliefs. Religion and spirituality was reported by the respondents as either a way of searching for health and protection or as a way of finding acceptance of their situation. There are also many family members, friends, and neighbours who are not afraid to be infected and who support and care for the people affected, for example:

*Interviewer: What kind of supporting did your husband gave it to you?*

*Woman: … Hmm like this: do not feel sad or do not feel discouraged… you are not alone. (FGD 18 People affected).*

## 6. Discussion

The findings regarding the meaning of leprosy show a confusion around the concepts *kusta* and *lepra *and an overall lack of knowledge of the illness, its causes, and ways of transmission. Moreover, the images and perceptions about leprosy already internalized or newly acquired give rise to feelings of fear. The perception that leprosy is a very infectious disease that can be transmitted by touching the same objects leprosy-affected persons have touched is worrisome. Hence, increasing knowledge about leprosy in people affected, community members and health workers remains an important goal for leprosy services, and although it not the sole answer to stigma, it is an essential prerequisite [35–37]. Several initiatives try to address this, for instance, the World Health Organisation has indicated key messages for the public and for people affected [38]. 

In addition, the importance of the darkening of the skin, an inevitable but temporary sideeffect of the MDT component clofazimine, should be underlined. This study indicated that it sometimes brought people in uncomfortable situations wherein they were questioned about their condition. It influenced their ideas about their own or others' beauty and cleanliness. A study in Brazil illustrated the connection made between the dark skin and racial ideas by this illustrative quote “Before, I was only a leper. But now, I'm going to be a nigger leper” [39]. This study also described the negative impact darkening of the skin can have on the intake of the medication. The topic is not yet well studied, but seems an important subject to improve leprosy services. 

Several challenges prevailed in relation to seeking care, recognizing the symptoms, making the correct diagnosis, sharing the diagnosis with the patient and the treatment. Noticeable is the power and influence of the leprosy workers and hence also the destructive impact of any stigmatizing behaviour from their side on the people in their care. White described this as “iatrogenic stigma” or stigma produced by a patient's encounter with health workers [40]. Here lies a great opportunity for the improvement of leprosy services for example through trainings. Since power is a dynamic concept it can be used in a positive manner as several leprosy workers already do. 

Quite a few respondents raised the aspect of sorcery as a cause of leprosy and consequently the role of the *dukun*. This was found in other countries such as Nepal, Nigeria, and Sudan [20, 41]. In the Indonesian context it makes sense to perceive them as relevant and valuable stakeholders for leprosy services, but again more research is needed on their knowledge, views, and approaches.

A recurring and thus overarching theme is “spirituality and religion.” It seems to help people affected by leprosy and community members give meaning (e.g., a challenge, punishment) to leprosy, as well as offer strategies to cope with the illness. Religion has been proved to play a major role for other stigmatised illnesses such as HIV/AIDS [42, 43]. This theme provides opportunities for leprosy services, but first raises questions regarding the current role of religious leaders and what role they could play in the future. Further research is needed that specifically addresses these questions.

It is clear that, according to the participants' life experiences, leprosy as a disease evokes feelings and experiences that are described as stigma [44]. On the side of the stigmatised it is easy to recognize the anticipated, internalized, and enacted stigma in the narratives from the participants. For example, in the reasons to discontinue school all three can be seen (i) embarrassed, ashamed and shy (internalized), (ii) peers making fun of them and taking distance (enacted), and (iii) fear of being insulted (anticipated). Likewise, the accepted, endorsed, and enacted stigmata perpetrated by stigmatisers are easy observable. Remarkable is that some people perceived shame and a low self-esteem as symptoms rather than a result of leprosy. This shows the solid ties between leprosy and stigma. It is important to note that stigma has lasting effects on people already declared cured according to WHO standards, even those without impairments or any other visible signs, as there are millions of people worldwide with this status. In spite of the fact that the current concept of stigma already comprises a variety of aspects, it is important for staff in leprosy or rehabilitation services to be aware of the wide range of experiences of people. Leprosy services need to be responsive to the diversity of experiences and the needs of people currently in treatment and those already cured. 

The two-way relationship between disability and poverty, that can become a vicious circle, has been extensively described [45, 46] and can be recognized in our narratives. Delayed health seeking due to poverty increases the risk of visible skin lesions and impairments, whereas our data show that having leprosy clearly has a negative influence on the socioeconomic status of a household, as also confirmed in other studies [47–49]. This is an important pointer for leprosy services to address. 

Although there are more negative than positive experiences, the positive ones are valuable as these can help stop reinforcing and potentially break down stereotypes and assumptions about leprosy. Leprosy could be seen as a paradoxical predictor of personal development. Positive experiences are, to our knowledge, sparsely described in the scientific literature. A notable exception is the STEP project that actively tried to transform the image of persons affected by leprosy to that of positive change agents [50, 51]. Fortunately there are some accounts in nonscientific literature such as (sometimes romanticized) autobiographies and a collection of publications to dignify and inspire [52–54].

Finally, subgroups that need specific attention are elderly and children, but also women and families. Little is written about these groups and their needs. We hope to address these sub-groups in more detail in another publication, but also encourage others to take these topics forward. 

## 7. Conclusion

The continued need for implementation of leprosy services in Indonesia is very evident, with a focus on early detection of new cases, prevention of impairments, and equal access for women. We conclude that the experiences of persons affected by leprosy, those under treatment and those that have completed treatment and have been declared cured, are diverse and go well beyond leprosy as an infectious disease or medical issue. A majority of respondents related strongly to the prevailing stereotypes about the disease and the related social stigma and discrimination. 

Leprosy services continue to be needed and should be strengthened and made more responsive to the diverse needs of affected persons where possible, including to some of those formally declared “cured”. In addition, this study emphasises the work that still needs to be done in terms of raising knowledge and awareness. Health workers need a greater understanding of their own power and sometimes stigmatizing behaviour. Specific interventions are needed to reduce stigma in the health services. The impact of the temporary darkening of the skin due to clofazimine should not be underrated. Other stakeholders in the community, such as religious leaders and traditional healers, need to be involved in attempts to reduce stigma. The impact of leprosy on the socioeconomic situation should not be underestimated. There is some evidence that interventions to improve people's socioeconomic status help to reduce stigma. This is currently being tested in the SARI Project. Positive images, experiences, and role models should be used to break down existing stereotypes depicting people affected by leprosy as pitiful and disgusting. Specific attention is needed for specific groups as children, elderly, families, and women. 

Leprosy services should take into account the wide diversity of meanings and experiences of people affected by leprosy and key persons in the community. This study shows that this can uncover important clues to make leprosy services more effective and appropriate.
